# Supplementary material for: Unveiling Low THz Dynamics of Liquid Crystals: Identification of Intermolecular Interaction among Intramolecular Modes
Source: J Phys Chem B. 2024 Jan 8;128(2):596–602. doi: 10.1021/acs.jpcb.3c07947 (PMC10801680; doi:10.1021/acs.jpcb.3c07947)
Supplement: Supplementary file 1 — jp3c07947_si_001.pdf [file jp3c07947_si_001.pdf]

Supplementary Information

Unveiling low THz Dynamics of Liquid Crystals: Identification of Intermolecular Interaction among Intramolecular Modes

Patrick Friebel[a], Daria Ruth Galimberti[b], Matteo Savoini[c], Laura Cattaneo[a*]

[a] Max Planck Institute for Nuclear Physics, Saupfercheckweg 1, 69117 Heidelberg, Germany
[b] Institute for Molecules and Materials, Radboud University, Heyendaalsweg 135, 6526 AJ Nijmegen, The Netherlands
[c] Institute for Quantum Electronics, ETH Zürich, Auguste-Piccard-Hof 1, 8093 Zürich, Switzerland

[*] Email: cattaneo@mpi-hd.mpg.de

Section A: Computational Details

A1. Conformational search summary

As stated in the main text, all the spectra discussed in the paper have been computed with the Gaussian16 code [1]. A conformational search was run using the Gaussview software [2] and the MMFF4 force field as a first step for all the analyzed cases, imposing an energy window of 3.5 kcal/mol. From the generated pool of structures, in the case of the single molecule calculations, all the conformations have been retained for the next step, while in the case of the 5CB dimer and trimer, due to the computational cost, the structures with an energy of more than 2 kcal/mol from the global minimum have been discarded. Table S1 reports the number of conformations found with these criteria and used to compute the vibrational for all the analyzed cases.

Table S1. Number of conformations used to compute the vibrational spectra

CB4 single molecule	7
CB5 single molecule	27
CB5 single dimer	119
CB5 single trimer	219
CB8 single molecule	264
PCH5 single molecule	29

A2 B3LYP vs. BLYP

Figure S1 compares the CB5 single molecule gas-phase harmonic spectra computed with the BLYP functional and the B3LYP functional (Gaussian16 code [18], 6-311++G** basis set, Grimme D3 dispersion term [3]).

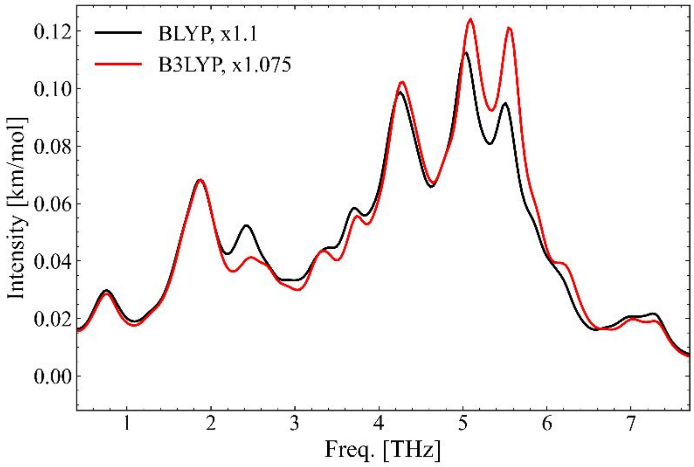

*Figure S1: BLYP-D3 (black) and B3LYP-D3 (red) 5CB harmonic spectra (6-311++G** basis set in both cases). The frequencies are scaled by different factors to match the experimental trace: BLYP-D3 by a factor 1.1, B3LYP-D3 by a factor 1.075. The spectra shown here have been obtained as a weighted average by the Boltzmann populations (at 323 K) of the set of computed spectra.*

The main difference between the two descriptions is a general shift of the modes in the BLYP spectrum compared to the B3LYP one. Apart from these, the BLYP functional and B3LYP functional predictions are similar for the spectral range we are considering. Therefore, in the case of the 5CB cluster calculations (dimers and trimers), the BLYP functional has been preferred to the more expensive B3LYP one.

A3. Comparison of the 5CB and 8CB absolute intensities

Figure S2 compares absolute intensities and the mode shapes of the 5CB and 8CB single molecule computed spectra.

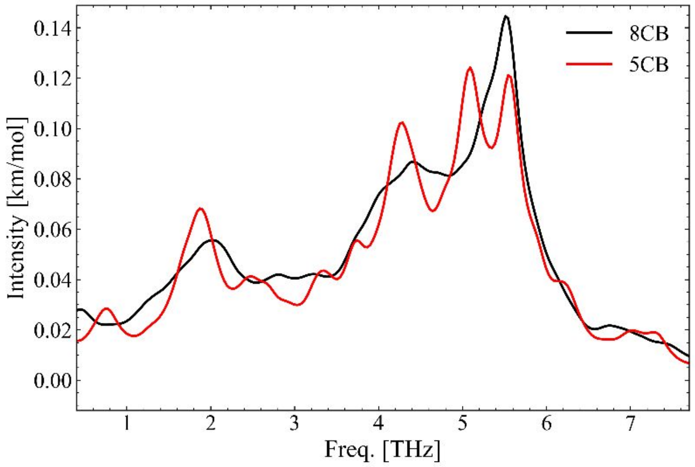

*Figure S2: B3LYP-D3/6-311++G** singe molecule harmonic spectra intensity for 5CB (red) and 8CB (black). The frequencies have been scaled by a factor 1.075. The spectra shown here have been obtained as a weighted average by the Boltzmann populations (at 323 K) of the set of computed ordinary spectra.*

5CB and 8CB have similar total (integrated) intensity per molecule in this spectral region. This is not surprising, considering that the activity of this region is dominated by the CN and the rings' contributions (charges and charges fluxes), and these groups are the same between the two molecules. However, the length of the tail still gives non-negligible effects on the spectra modulating the spectral appearance, in particular in the region above 4 THz. As discussed in the main text, the modes of this spectral region are always delocalized on the whole molecule and the number of CH_2 units modifies the couplings between ring and alkyl chain. In general, 5CB features are more narrow compared to the CB8 ones. This can also be observed in the experimentally measured isotropic phase spectra, albeit in a minor form due to the intermolecular interactions that broaden the features (Figure 3 of the main paper). Two modes are clearly visible between 5THz and 5.5THz for 4CB and 5CB, but not for 8CB and 10CB.

The region below 3.5THz instead shows in our computed spectra a broadening, but not dramatic changes between 5CB and 8CB. Therefore, 5CB has been considered a good enough model as a compromise for the computational cost to study the effects of the intermolecular interactions on this region for all the nCB.

A4. 5CB monomers, dimers, and trimers cluster calculations

The main document discussed only the low (1 - 3.5) THz mode (Figure 4, main text) when discussing the cluster calculations since this is the main point of disagreement with the experiment. The full calculated cluster spectra, zoomed out to the full range under investigation, are shown in Figure S3. It is evident, that in addition to the low THz mode, also the modes at higher frequency change.

The comparison of Boltzmann weighted computed spectra and the experiments above 4 THz seems not as good as in the case of 8CB already with the gas-phase molecule and get worse with the dimer and trimer clusters.

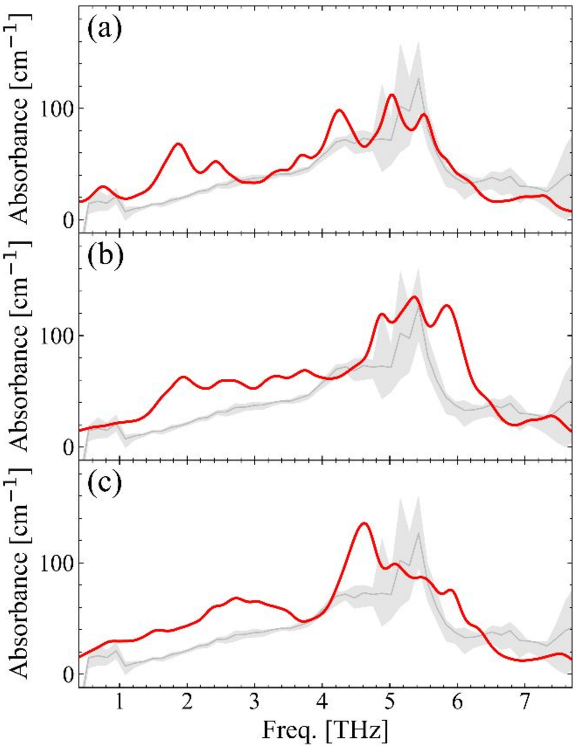

*Figure S3: Full traces of (a) Monomer, (b) Dimer, and (c) Trimer5CB cluster calculated harmonic spectra (BLYP-D3/6-311++G**). In each case the isotropic measurement result is included in the background (light grey) for additional comparison to the experiment. The computed intensities have been normalized on the strongest mode as in the main document. The frequencies have been scaled by a factor 1.1, as established in figure S1. The spectra shown here have been obtained as a weighted average by the Boltzmann populations (at 323 K) of the set of computed spectra.*

However, this letter effect is due to the artificial arrangement of the molecule in the clusters due to the gas-phase calculations that do not reflect the molecular order in the bulk LC condensed phase. The intermolecular arrangement is governed in this system by the balance between the (anisotropic) Van der Waals intermolecular interactions, the dipole interactions (that favor an antiparallel configuration), the rings $\pi - \pi$ stacking (that requires that the rings face each other). In a real bulk LC condensed phase, the molecules can orient themselves in antiparallel configurations without penalizing the Van der Waals interaction between the chain, and therefore they preferentially do it even in the isotropic phase [4].

Unfortunately, in small clusters instead these three driving forces are competitors. In the final balance between them, the antiparallel arrangement is partially (dimers) or totally (trimers) lost for most of the clusters.
To qualitative validate this hypothesis, instead of using the Boltzmann weights, we also computed average spectra using the degree of "antiparallelness" as weight (Figure S4). The degree of "antiparallelness" has been defined as:

$$w = \exp\left(-(\cos 180 - \cos\theta)\right)$$

for the dimers and

$$w = \exp\left(-(\cos 180° - \cos\theta_1)\right)\exp\left(-(\cos 180° - \cos\theta_2)\right)\exp\left(-(\cos 180° - \cos\theta_3)\right)$$

for the trimers, where θ_i is the angle between the rings of the two molecule, as depicted in figure S4.

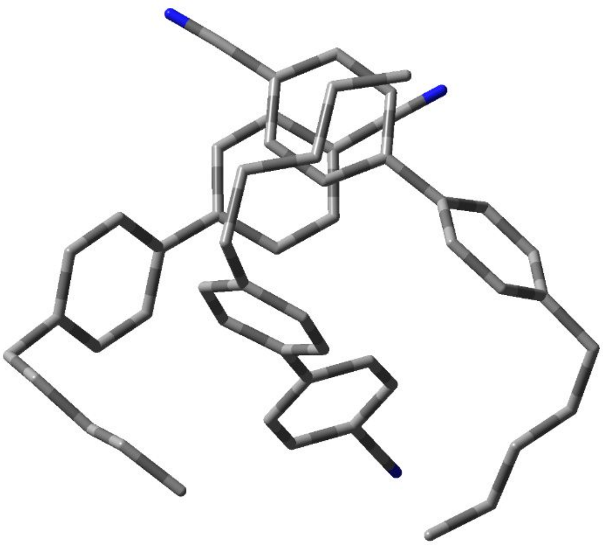

Figure S4: Example of a trimer cluster configuration

From Figure S5 one can see, that while the low THz mode remains largely unaffected, the high-frequency (4 - 6 THz) components are substantially modified (especially for the trimer case, since the dimer configuration already approaches the antiparallel arrangement by itself) towards closer resemblance with the experimental traces.

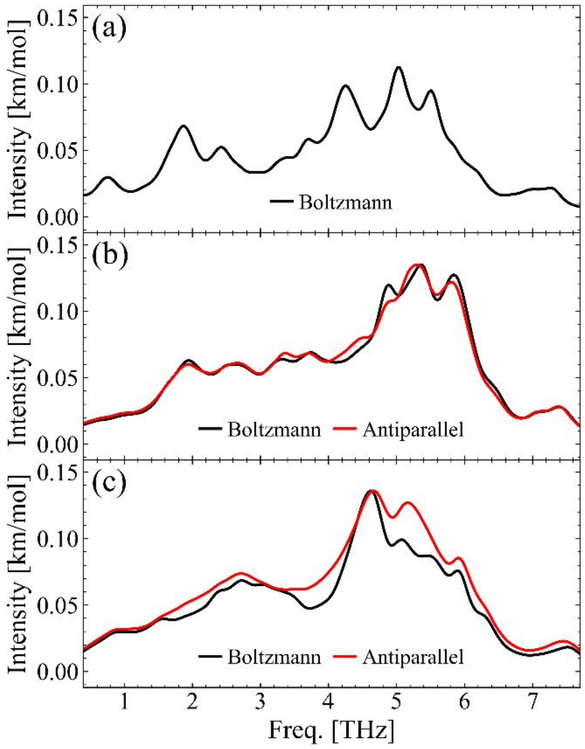

Figure S5:Boltzmann (black) and antiparallel weighting (red) distributions for the monomer (a), dimer (b), and trimer (c) cluster calculations at BYLP-D3 level. The Boltzmann results are given in absolute units, while the antiparallel traces are normalized to the maximum Boltzmann values. The frequencies are scaled by a factor 1.1.

Section B: Extraction Order Parameter S

We follow the procedure outlined in [5], with further reference to [6] [7]:

The order parameter S can be expressed as

$$S = \frac{n_e^2 - n_o^2}{\overline{n}^2 - 1} \frac{\overline{\alpha}}{\Delta\gamma}$$

where $n_{o/e}$ are the ordinary and extraordinary refractive indices, $\overline{n}^2 = \frac{1}{3}n_e^2 + \frac{2}{3}n_o^2$, $\overline{\alpha}$ is the mean polarizability, and $\Delta\gamma$ is the difference between longitudinal and transverse polarizability components of perfectly ordered molecules.

The refractive indices are a natural result of Time Domain Spectroscopy measurements and are shown in figure S6 for 8CB. We average the region form 2.8 - 3.2 THz, which is the region of interest for the comparison with the total absorption of the lowest absorption mode.

The ratio $\Delta\gamma/\overline{\alpha}$ can be extracted by extrapolation to 0 K of a sufficiently large data set. Here we restrict ourselves to extrapolation from the results of Vieweg et al. [5], who did follow the full procedure for 5CB, 6CB, and 7CB. We are aware of the limitation of this approach, but we consider it sufficient for establishing a general trend in S. For 8CB we choose $\Delta\gamma/\overline{\alpha} = 0.4 \pm 0.025$, with the error taking into account the inherent uncertainty in this method of extrapolation. The error of S has been computed by standard error propagation Furthermore, it must be stated that this approach has been developed for nematic liquid crystals, while we apply it across mesophases.

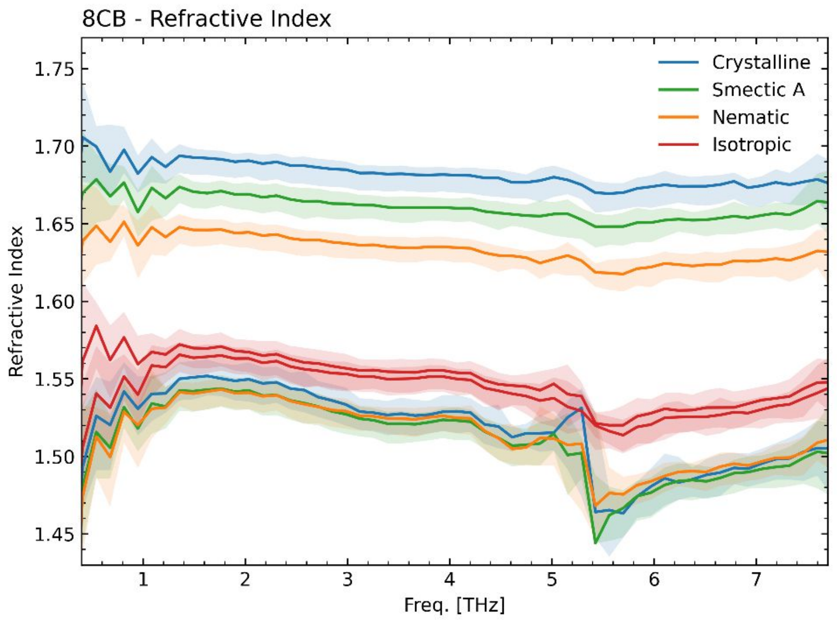

Figure S6: Refractive indices of 8CB for different Temperatures, as extracted from the same TDS data as used for the absorption traces shown in figure 2 in the main document.

Section C: Total intensity of individual absorption modes

The uncertainty bands (shaded area in Figure S7) associated with the TDS absorption spectra reflect the range of all measured traces while acquiring the data set. While the bold result is computed using the averaged reference and sample time-traces, we compute the result of all possible combinations of single unaveraged traces to estimate the uncertainty. By taking the maximum and minimum of this collection of results, we provide a guide to the eye for the confidence level. We stress that these results should not be taken as robust 95% confidence intervals or standard deviations. The increased level of uncertainty above 4 THz originates from the drop in the generated THz spectral power (compare Figure 1b in the main text).

We fit each absorption trace with a sum of 4 Lorenz curves. A representative example is shown in figure S7. In order to determine the intensity, the Lorentz line shapes are numerically integrated. For comparison, they are normalized to the isotropic case. While we focus on the lowest absorption mode, figure S8 shows the results of this approach for all modes of 8CB, and the total absorption consisting of all four modes.

The error bars linked to the total intensities are created in a Monte-Carlo style manner. For every fit parameter we generate 10000 normally distributed values, centered around the fit result with width of the error of the fit. Using these randomized line shapes, we take the standard deviation as the error bar plotted in the Abs. vs. S figures, i.e. Fig. S7 and S8.

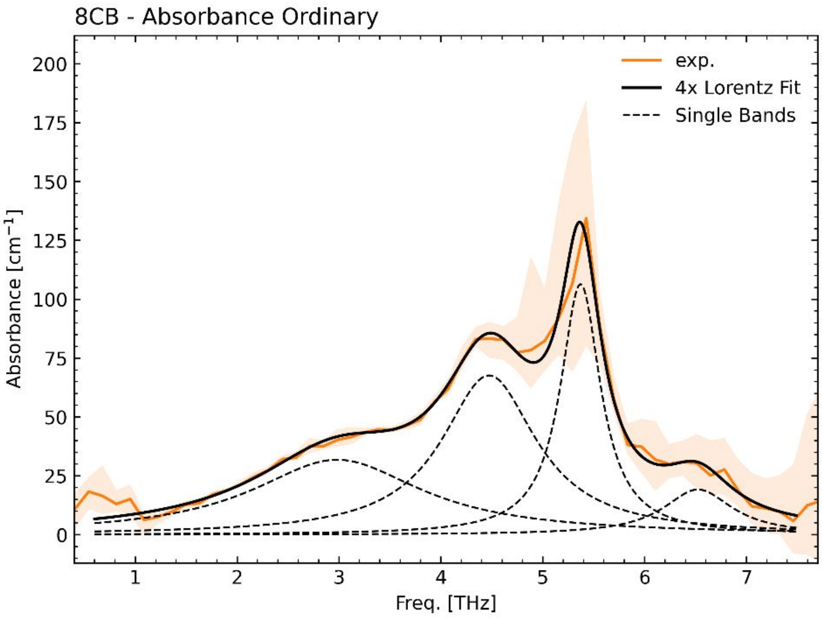

Figure S7: Fit of the absorption modes of 8CB, here for the nematic case as a representative example. The fit result is shown in solid black, the dashed curves are the individual modes extracted from the result.

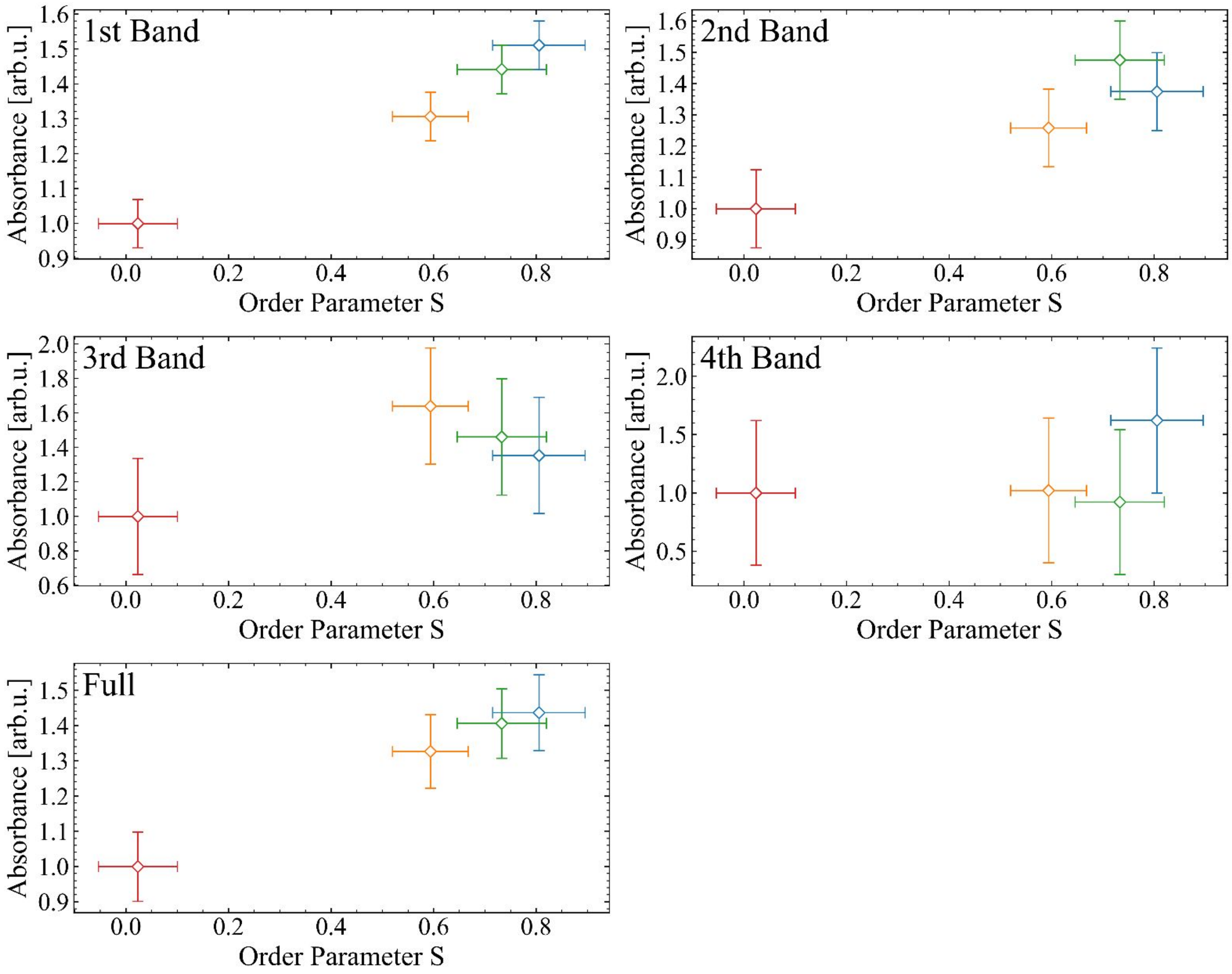

Figure S8: Results of the determination of the total intensity for all four modes and all four temperatures of 8CB, as a function of the order parameter, extracted as described in section B.

Section D: Details on liquid crystal compounds

Table S2: Liquid Crystal compounds under investigation. All LCs are commercially available from Synthon Chemicals. The phase transitions and purities are taken from the product information. Phases: Cr - crystalline, SmA - smectic A, N - nematic, I - isotropic

Sample	Full Name	Phase Transitions	Purity [%]
4CB	4-Butyl-4'-cyanobiphenyl	Cr - 48°C - I	99.5
5CB	4-Pentyl-4'-cyanobiphenyl	Cr - 24°C - N - 35.5 - I	99.5
8CB	4-Octyl-4'-cyanobiphenyl	Cr - 21.5°C - SmA - 33°C - N - 40.5°C - I	99.5
10CB	4-Decyll-4'-cyanobiphenyl	Cr - 44°C - SmA - 51.1 - I	99
PCH5	trans-4-(4´-n-Pentylcyclohexyl)benzonitrile	Cr - 31°C - N - 55°C - I	99.5

Section E: Experimental and Theoretical PCH5 response

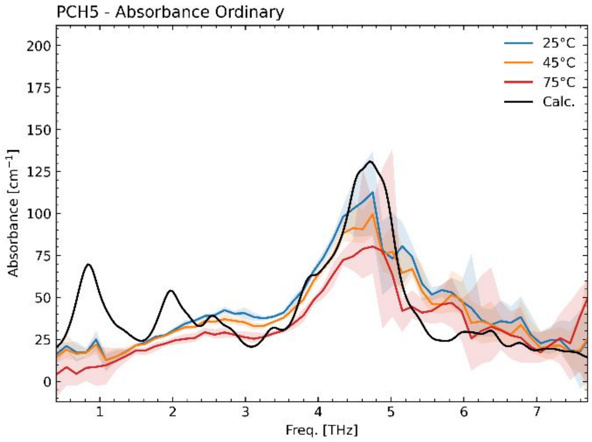

Figure S9: TDS Spectra for PCH5 for the ordinary (left) and extraordinary (right) cases. The Calculated results are overlaid in solid black lines.

References

[1] M. J. Frisch, G. W. Trucks, H. B. Schlegel, G. E. Scuseria, M. A. Robb, J. R. Cheeseman, G. Scalmani, V. Barone, G. A. Petersson, H. Nakatsuji, X. Li, M. Caricato, A. V. Marenich, J. Bloino, B. G. Janesko, R. Gomperts, B. Mennucci, H. P. Hratchian, J. V. Ortiz, A. F. Izmaylov, J. L. Sonnenberg, Williams, F. Ding, F. Lipparini, F. Egidi, J. Goings, B. Peng, A. Petrone, T. Henderson, D. Ranasinghe, V. G. Zakrzewski, J. Gao, N. Rega, G. Zheng, W. Liang, M. Hada, M. Ehara, K. Toyota, R. Fukuda, J. Hasegawa, M. Ishida, T. Nakajima, Y. Honda, O. Kitao, H. Nakai, T. Vreven, K. Throssell, J. A. Montgomery Jr., J. E. Peralta, F. Ogliaro, M. J. Bearpark, J. J. Heyd, E. N. Brothers, K. N. Kudin, V. N. Staroverov, T. A. Keith, R. Kobayashi, J. Normand, K. Raghavachari, A. P. Rendell, J. C. Burant, S. S. Iyengar, J. Tomasi, M. Cossi, J. M. Millam, M. Klene, C. Adamo, R. Cammi, J. W. Ochterski, R. L. Martin, K. Morokuma, O. Farkas, J. B. Foresman and D. J. Fox, *Gaussian 16 Rev. C.01,* Wallingford, CT, 2016.

[2] R. Dennington, T. A. Keith and J. M. Millam, *GaussView Version 6.1,* 2016.

[3] S. Grimme, J. Antony, S. Ehrlich and H. Krieg, "A consistent and accurate ab initio parametrization of density functional dispersion correction (DFT-D) for the 94 elements H-Pu," *The Journal of Chemical Physics,* vol. 132, April 2010.

[4] S. Takabatake and T. Shikata, "Evidence of anti-parallel dimer formation of 4-cyano-4′-alkyl biphenyls in isotropic cyclohexane solution," *Physical Chemistry Chemical Physics,* vol. 17, p. 1934–1942, 2015.

[5] N. Vieweg, C. Jansen, M. K. Shakfa, M. Scheller, N. Krumbholz, R. Wilk, M. Mikulics and M. Koch, "Molecular properties of liquid crystals in the terahertz frequency range," *Optics Express,* vol. 18, p. 6097, March 2010.

[6] M. F. Vuks, "Determination of the optical anisotropy of aromatic molecules from the double refraction of crystals," *Optics and Spectroscopy,* vol. 20, p. 361, 1966.

[7] I. Haller, "Thermodynamic and static properties of liquid crystals," *Progress in Solid State Chemistry,* vol. 10, p. 103–118, January 1975.
